# The Free Radical Scavenger N-Tert-Butyl-α-Phenylnitrone (PBN) Administered to Immature Rats During Status Epilepticus Alters Neurogenesis and Has Variable Effects, Both Beneficial and Detrimental, on Long-Term Outcomes

**DOI:** 10.3389/fncel.2018.00266

**Published:** 2018-08-28

**Authors:** Hana Kubová, Jaroslava Folbergrová, Jana Rejchrtová, Grygoriy Tsenov, Martina Pařízková, James Burchfiel, Anna Mikulecká, Pavel Mareš

**Affiliations:** ^1^Department of Developmental Epileptology, Institute of Physiology of the Czech Academy of Sciences, Prague, Czechia; ^2^Strong Epilepsy Center, Department of Neurology, University of Rochester Medical Center, Rochester, NY, United States

**Keywords:** juvenile rats, epilepsy, epileptic comorbidities, adult neurogenesis, free radical scavenger, neuroprotection

## Abstract

Status epilepticus (SE), especially in immature animals, is known to produce recurrent spontaneous seizures and behavioral comorbidities later in life. The cause of these adverse long-term outcomes is unknown, but it has been hypothesized that free radicals produced by SE may play a role. We tested this hypothesis by treating immature (P25) rats with the free radical scavenger N-tert-butyl-α-phenylnitrone (PBN) at the time of lithium chloride (LiCl)/pilocarpine (PILO)-induced SE. Later, long-term outcomes were assessed. Cognitive impairment (spatial memory) was tested in the Morris water maze (MWM). Emotional disturbances were assessed by the capture test (aggressiveness) and elevated plus maze’s (EPM) test (anxiety). Next, the presence and severity of spontaneous seizures were assessed by continuous video/EEG monitoring for 5 days. Finally, immunochemistry, stereology and morphology were used to assess the effects of PBN on hippocampal neuropathology and neurogenesis. PBN treatment modified the long-term effects of SE in varying ways, some beneficial and some detrimental. Beneficially, PBN protected against severe anatomical damage in the hippocampus and associated spatial memory impairment. Detrimentally, PBN treated animals had more severe seizures later in life. PBN also made animals more aggressive and more anxious. Correlating with these detrimental long-term outcomes, PBN significantly modified post-natal neurogenesis. Treated animals had significantly increased numbers of mature granule cells (GCs) ectopically located in the dentate hilus (DH). These results raise the possibility that abnormal neurogenesis may significantly contribute to the development of post-SE epilepsy and behavioral comorbidities.

## Introduction

It is well known that status epilepticus (SE) results in the development of recurrent seizures and behavioral comorbidities. Researchers have identified numerous changes in the brain following SE that may contribute to these outcomes. Prominent among these changes are neuropathological modifications within the hippocampus, including the loss of specific neuronal populations and abnormal neurogenesis, especially the aberrant migration of newborn granule cell (GC) neurons to the dentate hilus (DH).

The mechanisms underlying these neuropathological changes, however, remain largely unknown. Recently, increased attention has been paid to the role of free radicals in epileptogenesis. Seizures are known to produce excessive free radicals, which in turn can substantially contribute to seizure-induced neuronal damage in both adult (Bruce and Baudry, 1995; Ueda et al., 2002) and developing animals (Patel and Li, 2003; Folbergrová et al., 2006, 2016).

If free radicals play a role in neuropathological damage following SE, then it follows that neutralizing these agents during SE might be able to reverse or substantially ameliorate this damage. We tested this hypothesis using the free radical scavenger N-tert-butyl-α-phenylnitrone (PBN). We chose this agent because of its demonstrated acute neuroprotective effects in many neurodegenerative models (for review see Hensley et al., 1997) including different seizure models in both adult (He et al., 1997; Folbergrová et al., 1999) and immature (Folbergrová et al., 2006) rats. Past studies from our laboratory have had similar results. Juvenile (P25) rats treated with PBN during lithium chloride (LiCl)/pilocarpine (PILO)-induced SE demonstrated a decrease of acute neurodegeneration in several hippocampal areas and improvement of functional recovery after SE (Rejchrtová et al., 2005). It is not known, however, whether these short-term favorable outcomes are permanent.

The aim of the present study was to investigate the long term outcomes following PBN administration at the time of SE in juvenile rats. We looked at the effect of this treatment on the development of chronic epilepsy later in life and on behavioral co-morbidities. We began with the hypothesis that PBN treatment would provide long-lasting protection.

As sometimes occurs in scientific investigation, our results were surprising. Instead of the neuroprotective effects seen acutely, PBN’s long-term effects were mixed. PBN significantly ameliorated some of the long-term harmful effects of SE, but exacerbated other effects, most notably chronic seizures. Moreover, we found an intriguing correlation between this exacerbation of chronic epilepsy and aberrant neurogenesis in the hippocampus. This finding provides major support for a new hypothesis that aberrant neurogenesis may be a significant mechanism underlying the development of chronic epilepsy following SE.

## Materials and Methods

### Animals

Experiments were performed in male Wistar albino rats (Institute of Physiology of the Czech Academy of Sciences). The day of birth was defined as day 0 and animals were weaned at P21. Rats were housed in a controlled environment (temperature 22 ± 1°C, humidity 50%–60%, lights on 6 am–6 pm) with free access to food and water. All procedures involving animals and their care were conducted according to the ARRIVE guidelines^1^ in compliance with national (Act No 246/1992 Coll.) and international laws and policies (EU Directive 2010/63/EU for animal experiments and the National Institutes of Health guide for the care and use of Laboratory animals (NIH Publications No. 8023, revised 1978). The experimental protocol was approved by the Ethical Committee of the Czech Academy of Sciences (Approval No. 128/2013).

### Induction of Status Epilepticus

The animals were pretreated at 24 days of age (P24) with LiCl 127 mg/kg i.p. Each animal was assigned a code, so its individual history could be maintained throughout the entire study. The next day (P25), SE was induced by a single dose of PILO 40 mg/kg i.p.

The onset of SE was defined as the appearance of clonic motor seizures. In order to decrease mortality, a single dose of paraldehyde (0.3 ml/kg i.p.; Fluka Chemie AG, Buchs, Switzerland) was injected 2 h after the onset of SE (for details see Kubová et al., 2005).

The latency to the onset of motor seizures and mortality were recorded. Only rats that exhibited behavioral manifestations of seizures progressing to forelimb clonus for at least 1 h, without periods of wild running and generalized tonic-clonic seizures, were used for further studies. It was important that the experimental groups had this uniform level of SE severity because our previous study (Rejchrtová et al., 2005) demonstrated that PBN pretreatment suppresses the progression to generalized tonic clonic seizures.

After paraldehyde injection, the rats were subcutaneously injected with 0.9% NaCl (up to 3% of the body weight divided into 2–3 doses) to restore volume loss. For about 3–4 days after SE, animals were fed a moist diet. Their health status was monitored daily until the end of study.

### Treatment

PBN (#B/7263-5G, Sigma Chemical Co., St. Louis, MO, USA) was administered at a total dose of 200 mg/kg, i.p. (freshly dissolved in saline, 10 mg/ml), divided into two consecutive doses as detailed below. This regimen is based on previous studies by others (Kotake, 1999) and our own experience (Rejchrtová et al., 2005).

#### Treatment Groups

- *SE group* (*n* = 12): these animals were subjected to PILO induced SE without PBN treatment. Instead of PBN, these animals received a corresponding volume of saline.
- *PBN/PILO group* (*n* = 10): these animals received two doses of PBN (100 mg/kg i.p. each) in association with PILO: one 30 min prior to PILO and the other 1 min after SE onset.
- *PILO/PBN group* (*n* = 13): these animals also received two doses of PBN (100 mg/kg i.p. each) in association with PILO, but the timing was different from the PBN/PILO group. The first dose was given 1 min after SE onset and the other 60 min later.

#### Control Groups

- *C group* (*n* = 10): these animals received the same treatment as animals in the SE group except that PILO was replaced with an equal volume of saline. Comparison of this group to the SE group determined the effects of SE alone.
- *PBN group* (*n* = 10): these animals also received an equal volume of saline instead of PILO. Otherwise the treatment was the same as in PBN treated animals exposed to SE. Comparison of this group to the animals treated with PBN and SE determined the effects of PBN alone.

**Figure 1:**
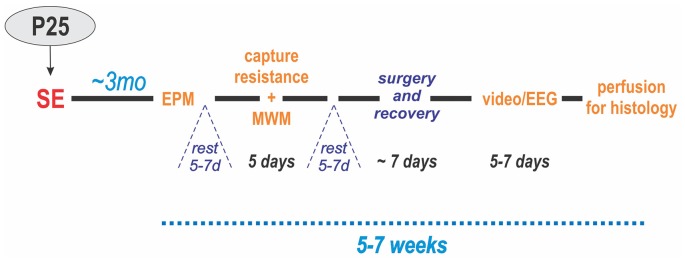
Experimental design. Status epilepticus (SE) was induced at P25. Three months later, the animals were administered behavioral tests in two blocks: (1) elevated plus maze (EPM) and (2) resistance to capture and Morris water maze (MWM). After each block, the animals were allowed to rest for 5–7 days. Next, electrodes were implanted in the hippocampus and cerebral cortex of the left hemisphere followed by 1 week of recovery. Then, they underwent continuous video/EEG monitoring for 5–7 days. Lastly, the animals were perfused for histology.

The experimental design of the study for these groups is illustrated in Figure 1.

### Assessment of Behavioral Parameters in Adulthood

Behavioral testing of each animal was started 3 months after SE. For the elevated plus maze (EPM) and the Morris water maze (MWM), testing was done in a special room with constant temperature (22 ± 1°C) and light conditions. Before testing, animals were allowed to adapt to the testing room for at least 2 h. All tests were performed between 10:00 h and 14:00 h. Behavior of the animals during testing was video recorded and analyzed off-line by two experienced observers.

#### Elevated Plus Maze

**Figure 2:**
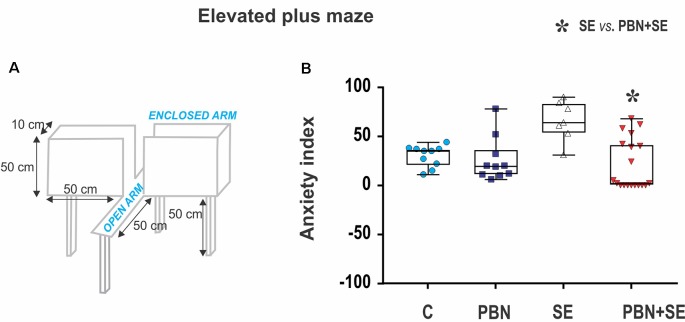
Long term effect of N-tert-butyl-α-phenylnitrone (PBN) treatment on behavior in the EPM. **(A)** Schematic of the EPM. **(B)** Anxiety index scores for individual animals in each group. Individual values are plotted over box plots (median with 25%–75%) with whiskers indicating minimal and maximal values. SE increases the anxiety index (i.e., percentage of time spent in open arms) and this effect is reversed by PBN. The PBN+SE animals actually appear to be more anxious with most of them clustering at or near the maximum score. (Abbreviations: C, control group receiving neither SE nor PBN; PBN, control group receiving PBN but not SE; SE, experimental group receiving only SE; and PBN+SE, experimental group treated with PBN at the time of SE. Symbols: Blue circles—C, dark blue squares—PBN, white triangles—SE, red triangles—PBN+SE).

Anxiety was assessed using the EPM (Figure 2A). For details see Kubová et al. (2004). Briefly, at the beginning of the experiment, the rat was placed onto the central square facing an open arm and was allowed to freely explore the maze for 5 min. The rat was considered to have entered an arm when all four limbs were inside the arm. The time spent in open or enclosed arms, respectively, was recorded. The level of anxiety (*anxiety index*) was calculated for each animal as the percentage of time spent in the open arms.

#### Resistance to Capture

Aggressiveness was assessed by resistance to capture. This test was performed before the first training session in the MWM. The test took place in the animal room as the rats were being transferred to new cages for transport to the room containing the water pool. It should be noted that about 1 week previously the animals had been handled during testing in the EPM. At that time, the animals were housed in their home cages in the testing room, and they were moved gently to the maze by holding them by the tail.

Resistance to capture was assessed in a single trial during which the animals were grasped around the chest. Each animal was assigned a score reflecting its most severe behavioral reaction according to the following scale (adapted from Pinel et al., 1977):

1 - Remains calm when grasped—easy to handle;
2 - Avoids hand by running—resists capture;
3 - Hyperactivity—leaps to avoid capture, and struggles vigorously when captured;
4 - Extreme hyperactivity—leaps, struggles and bites when captured, aggressive toward siblings sharing home cage (i.e., necessary to house animals individually).

#### Morris Water Maze

Spatial memory was assessed in the MWM. The parameters of the water maze are illustrated in Figure 4A. The method has been detailed previously (Kubová et al., 2004). The escape platform was placed 1 cm below the water surface and its position was kept the same throughout the experiment.

**Figure 3:**
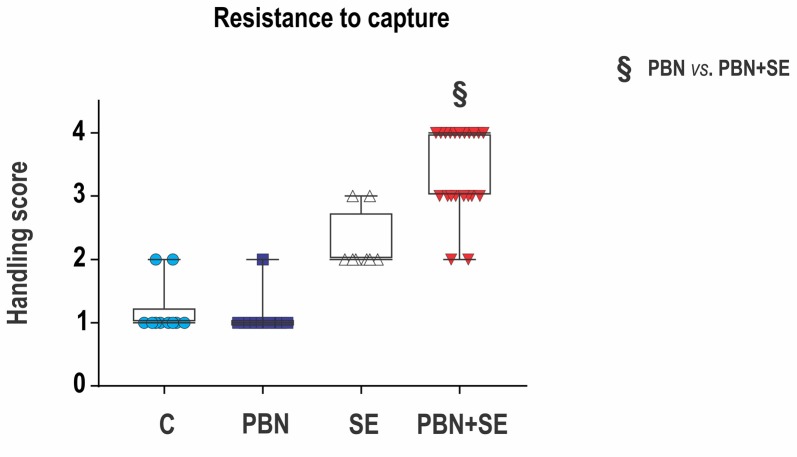
Long term effect of PBN treatment on resistance to capture. SE resulted in a significant increase of aggressiveness. PBN treatment during SE produced even greater aggressiveness. Capture scores of individual animals are plotted over box plots (median with 25%–75%) with whiskers indicating minimal and maximal values. (Abbreviations the same as in Figure 2).

**Figure 4:**
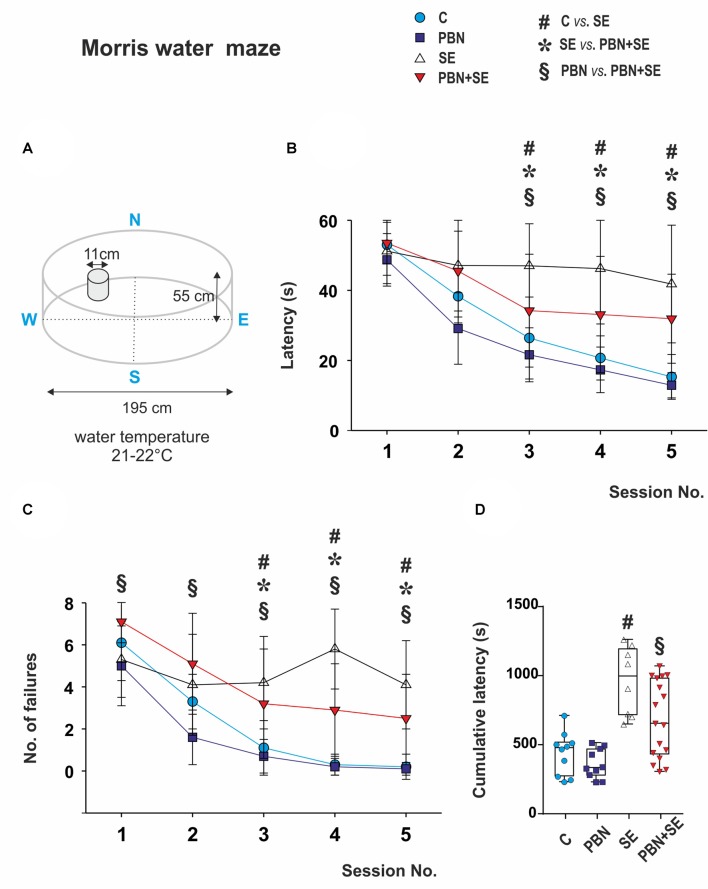
Long term effect of PBN treatment on performance in the MWM. **(A)** Schematic of the MWM. **(B)** Mean latencies ± standard deviation (SD) to reach the escape platform on each of the testing days for each animal group. SE animals were unable to learn the task. PBN treatment partially reversed this effect. PBN+SE animals performed significantly better than SE animals but not as well as either C or PBN animals. **(C)** Number of unsuccessful trials on each of the 5 days of testing for each group. Starting at day 3, animals treated with PBN during SE had significantly fewer failures than did the SE animals. Both groups, however, performed worse than the control animals without SE. **(D)** Cumulative latencies to reach the escape platform (days 3–5) for individual animals in each group. Data presented as box plots (median with 25–75 percentiles) with whiskers indicating minimal and maximal values (Abbreviations the same as in Figure 2).

Each rat received one training session (eight 60 s trials) per day for five consecutive days. The escape latency was measured for each trial. If the animal failed to find the escape platform, the trial was recorded as a failure and given a latency of 60 s.

To characterize the best performance reached by each animal, a *cumulative latency* was calculated by adding all latencies from days 3, 4 and 5. At this point all groups had reached asymptotic performance.

### Video/EEG Monitoring of Spontaneous Seizure Activity in Adulthood

After behavioral testing was finished (approximately 4 months after SE), an epidural silver ball electrode (AP = 0; *L* = 2.5 mm) and an intrahippocampal stainless steel electrode (AP = −3.5; *L* = 3; *H* = 3.5 mm) were implanted as described before (Kubová and Mareš, 2013) into the left hemisphere. The right hemisphere was preserved for later histological analyses. Animals were allowed to recover for about 1 week and then video/EEG was monitored continuously (24 h/day) for 5–7 days.

Video/EEG recording and off-line analyses were performed as described previously (for details see Kubová et al., 2004). An electrical seizure was defined as an episode of at least 5 s of rhythmic sharp elements. The following parameters were measured: (1) the mean number of ictal electrographic seizures per 24 h (seizure frequency); (2) the mean seizure duration; and (3) the total amount of time spent in seizures per 24 h (seizure activity). For every electrical seizure, the corresponding video recording was checked for the presence of convulsions. Recordings were analyzed by two experienced professionals.

In addition to formal video/EEG monitoring, throughout the study, animals were observed daily for up to 30 min during regular checks of their status in the animal room. Any behavioral seizures seen during these times were noted.

### Histology

Within 1 week after completing the video/EEG monitoring, rats were overdosed with an intraperitoneal injection of urethane (2 g/kg, Sigma Chemicals), transcardially perfused with 4% paraformaldehyde and processed for histology as described before (for details see Kubová et al., 2004). Adjacent series of frozen sections (1-in-5, 50 μm) were used for the following: (1) cresyl violet staining; (2) immunohistochemistry for doublecortin (DCX); (3) immunohistochemistry for prospero-like homeobox protein 1 (Prox1); and (4) double-label immunohistochemistry to detect co-localization of Prox1 and the neuronal specific nuclear protein (NeuN).

### Assessment of Hippocampal Morphology in Adulthood

Analysis of hippocampal morphology was performed using methods previously described and verified with magnetic resonance imaging (MRI; Nairismägi et al., 2006). Briefly, to assess hippocampal thickness, a stack of three consecutive cresyl violet stained sections (250 μm apart) was used for analysis. Measurements were started at the level corresponding to antero-posterior level −3.30 from Bregma according to the rat brain atlas of Paxinos and Watson (1986). Three lines were drawn between the alveus and the ventral surface of the infrapyramidal molecular layer (Figure 6A). The length of each line defined the thickness of the hippocampus at that point. This process was repeated for each of the three sections. The mean of all measurements per animal was calculated and used for statistical analysis.

**Figure 5:**
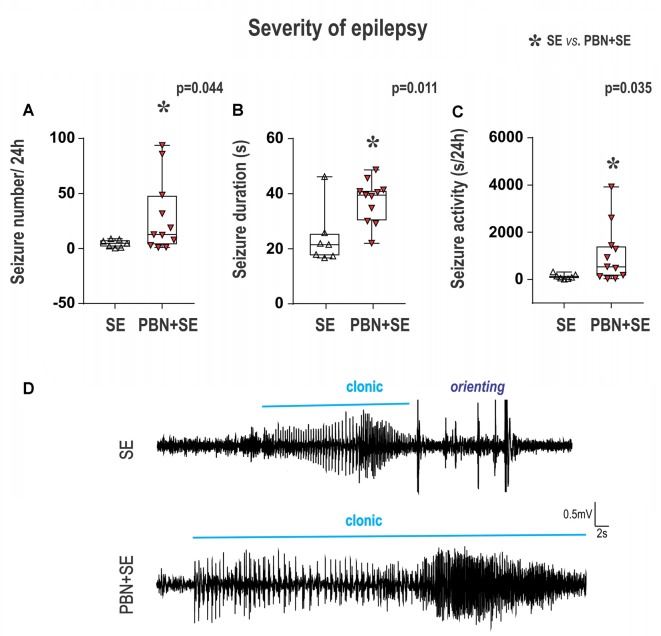
Long term effect of PBN treatment on seizure parameters of chronic epilepsy. Surprisingly, animals treated with PBN at the time of SE had significantly more severe chronic epilepsy as measured by seizure frequency **(A)**, seizure duration **(B)** and seizure activity **(C)**. Data presented as box plots (median with 25–75 percentiles) with whiskers indicating minimal and maximal values. In the lower half of the figure **(D)** are representative examples of hippocampal EEG recordings of seizures for SE and PBN+SE animals, respectively. Clonic motor behavior is indicated by the horizontal line above each tracing. Orienting = an intense orienting reaction (Abbreviations the same as in Figure 2).

**Figure 6:**
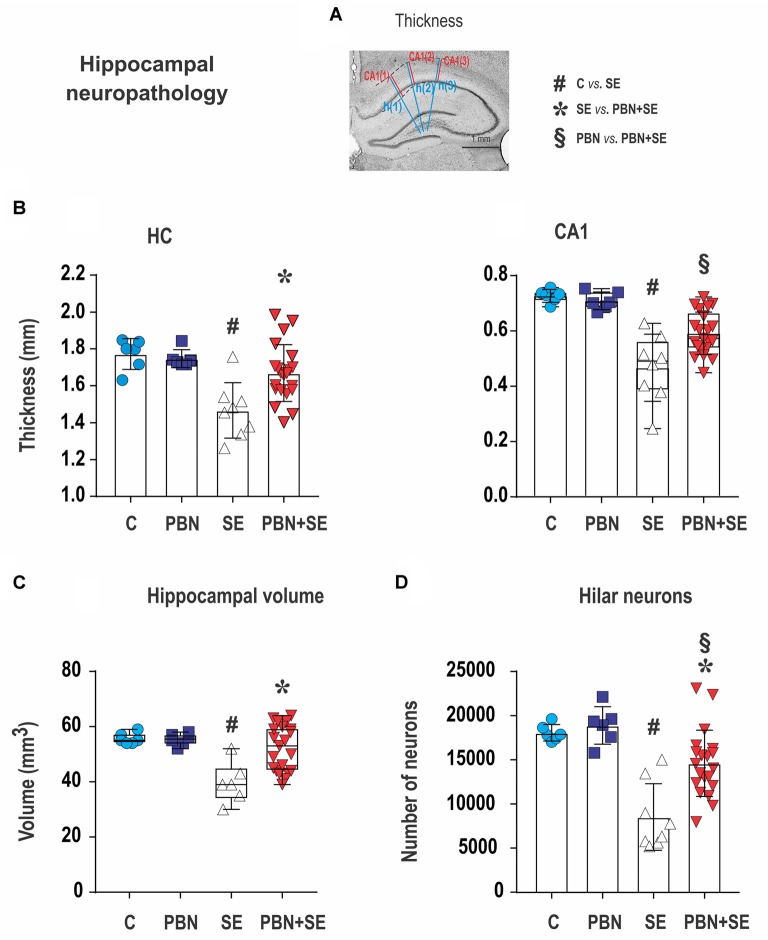
Long term effect of PBN treatment on hippocampal neuropathology. **(A)** Cresyl violet stained coronal section of the hippocampus illustrating how measurements of thickness were performed. The three blue lines [h(1) – h(3)] were used to measure overall hippocampal thickness. Adjacent red lines [CA(1) – CA(3)] were used for measuring the thickness of the CA1 subregion. See “Materials and Methods” section for details. The other four panels show hippocampal measurements for individual animals in each group. **(B)** Left panel shows that hippocampal (HC) thickness was significantly reduced by SE and this effect was reversed by PBN treatment. Right panel shows that the thickness of the CA1 subregion was significantly reduced by SE and this effect was partially reversed by PBN treatment. The PBN+SE group had a significantly thicker CA1 than the SE group but was still thinner than the control group. Data presented as a mean with SD. **(C)** Hippocampal volume was significantly reduced by SE and this effect was reversed by PBN treatment. Data presented as box plots (median with 25–75 percentiles) with whiskers indicating minimal and maximal values. **(D)** Unbiased stereological counts of neurons in the dentate hilus (DH) for individual animals in each group. The number of neurons was significantly reduced by SE and this effect was partially reversed by PBN treatment. The number of cells in the PBN+SE group was significantly greater than in the SE group, but was still less than in the control group. Data represented as a mean ± SD (Abbreviations the same as in Figure 2).

CA1 thickness was measured separately as the distance between the alveus and the hippocampal fissure with lines drawn adjacent to those used for measurement of hippocampal thickness (Figure 6A).

The volume of the hippocampus (except for the pre- and parasubiculum) was estimated stereologically using all Nissl stained sections containing the hippocampal formation (250 μm apart). The method, based on the Cavalieri principle (Gundersen et al., 1988), has been described in detail previously (Kubová and Mareš, 2013). For each animal, the coefficient of error (CE) was calculated to assure sufficient accuracy of the estimate (CE < 0.05).

### Assessment of Hippocampal Neurogenesis in Adulthood

Neurons were counted stereologically using optical fractionators (MBF Bioscience, Williston, VT, USA), as described before (Kubová and Mareš, 2013). Counting was performed with a 100× oil immersion objective lens. The sections were randomly selected, beginning at the level corresponding to antero-posterior level −3.30 mm from Bregma (Paxinos and Watson, 1986) and continuing through the whole rostro-caudal extent of the hippocampus. The counting frame grid was applied randomly. The thickness of the slices was measured with each count. “Guard zones” of 2 μm were used to avoid abnormalities of the tissue surface (Dorph-Petersen et al., 2001). Only neurons that came into focus by passing from one optical plane to the next were counted. The cell counts were then calculated using the fractionator formula, taking into account section, area and thickness of sampling fractions (West et al., 1991). The area of the counting frames was slightly modified for each staining according to a preliminary test (see details below).

The number of hilar neurons (mossy cells and interneurons) was estimated in 10 Nissl-stained sections of the dorsal hippocampus using counting frames with an area of 1,444 μm^2^ and 104-μm x and y steps as described before (Kubová and Mareš, 2013).

For counting immunostained cells, the area of the counting frame was slightly modified for each staining based on the results of preliminary tests.

#### DCX Labeled Cells

Newborn neurons were detected with an antibody against DCX (goat polyclonal, dilution 1:500, detects C-terminus of DCX; Santa Cruz Biotechnology Inc., Santa Cruz, CA, USA), using the avidin-biotin method described previously in detail (Tuunanen et al., 1996).

The number of cells expressing DCX (DCX-ir cells) was assessed in the dentate GC layer (GCL) plus the dentate subgranular zone (SGZ), a region about two cells thick extending from the inner margin of the GCL. In addition, DCX-ir cells were assessed separately in the DH. The counting frame had an area of 2,500 μm^2^ and 64-μm x and y steps were used.

#### Prox1 Labeled Cells and Prox1/Neun Double Labeled Cells

To identify ectopic cells in the DH, sections were processed using nickel-enhanced diaminobenzidine peroxidase immunohistochemistry with an antibody raised against Prox1 (1:25,000; rabbit anti-Prox1 polyclonal antibody, Chemicon International Inc., Temecula, CA, USA) as described before (McCloskey et al., 2006). Prox1/NeuN double-labeling was performed following the method described by Myers et al. (2013).

The numbers of cells expressing Prox1 (Prox1-ir cells) and both Prox1 and NeuN (Prox1/NeuN-ir cells) were assessed in 10 sections using the method described by McCloskey et al. (2006). The neuronal number was determined using a counting frame of 1,600 μm^2^ and 104 μm x and y steps. GCs were defined as homogeneously darkly labeled spheres. Cells were counted as double-labeled only if they met the two following criteria: (1) the Prox1 positive nucleus and NeuN positive cytoplasm were brought into focus simultaneously at 100× magnification; and (2) the cytoplasm was clearly distinguishable. These criteria are demonstrated in Figure 8 and have been reported before (Myers et al., 2013).

**Figure 7:**
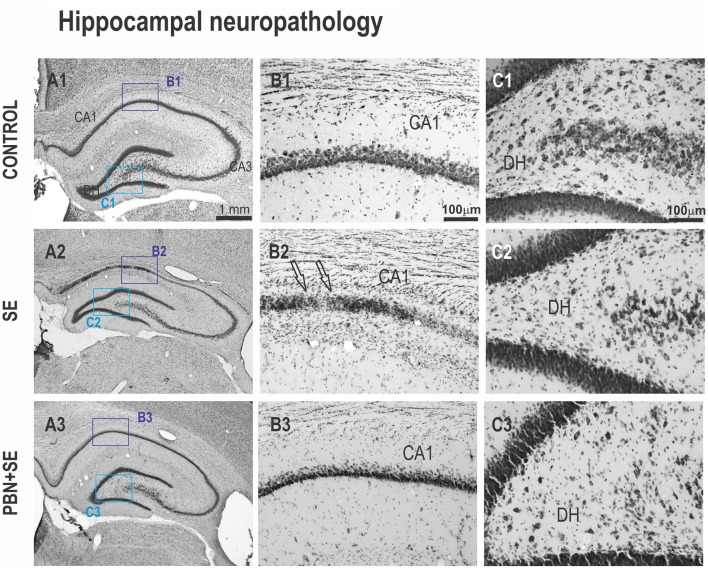
Long term effect of PBN treatment on hippocampal morphology. Left panels **(A1–A3)** show representative brightfield photomicrographs of Nissl stained sections of the hippocampus from control, SE and PBN+SE animals, respectively. **(B1–B3)** Panels show higher magnifications of the CA1 subregion. **(C1–C3)** Panels show higher magnifications of the DH. The regions of these magnifications are specified by the boxes in the **(A)** panels. In both CA1 and DH, the SE group shows extensive neuronal loss. Note the striking cell loss caused by SE in CA1 indicated by the arrows in **(B2)**. Significantly less damage is evident in the PBN+SE group **(B3,C3)**, but there are still fewer cells than in the control group.

**Figure 8:**
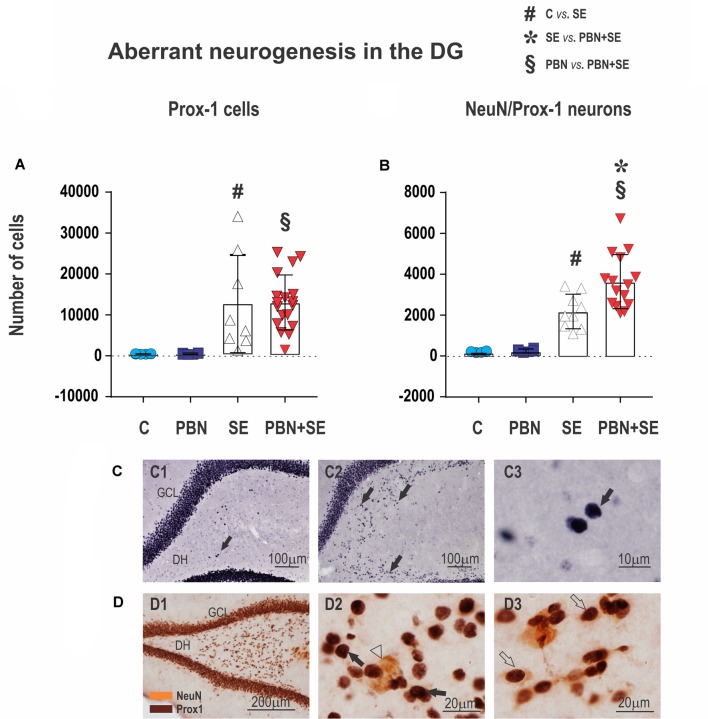
Long term effect of PBN treatment on aberrant neurogenesis in the dentate gyrus (DG). Upper panels show the results of unbiased stereological counts of neurons in the DH for individual animals in each group. **(A)** Cells labeled with an antibody to prospero-like homeobox protein 1 (Prox1). Both SE and PBN+SE animals show statistically increased numbers of Prox1-ir cells, but there is no difference between these groups. **(B)** Cells double-labeled with antibodies to Prox1 and neuronal specific nuclear protein (NeuN). In contrast to cells labeled only with Prox1, the number of double-labeled cells is statistically greater in the PBN+SE animals than in the SE animals not treated with PBN. **(C)** Representative photomicrographs of Prox1-ir cells ectopically located in the DH (black arrows). Only sparse Prox1-ir cells are observed in controls **(C1)**, whereas in animals experiencing SE, there are significantly more labeled cells **(C2)**. **(C3)** shows a compactly labeled cell at higher magnification. **(D)** Representative photomicrographs of aberrant cells in the DH labeled for Prox1 (brown stain) and NeuN (yellow stain). **(D1)** Low magnification. **(D2)** Higher magnification showing cells labeled for Prox1 (black arrow) and NeuN (white arrowhead). **(D3)** Cells doubled-labeled for Prox1 and NeuN (white arrows). In these double-labeled cells, the Prox1 labeling in the nucleus and the NeuN labeling in the cytoplasm come into focus in the same plane. Data represented as a mean ± SD (Abbreviations: DH, dentate hilus; GCL, granule cell layer. Other abbreviations same as in Figure 2).

### Statistical Analysis

Sample size was determined in advance according to previous experience with the given model and followed the principles of the three R’s (Replacement, Reduction and Refinement^2^). Outcome measures and statistical tests were prospectively selected. At the beginning of the study, a simple randomization was used to assign each animal to a particular treatment group. Data acquisition and analysis were done blinded to the treatment.

The data were analyzed using GraphPad Prism 7 (GraphPad Software, La Jolla, CA, USA) software. Using the D’Agostino and Pearson normality test, all data sets were first analyzed to determine if the values are derived from a Gaussian distribution. Based on these results, it was determined whether to use nonparametric or parametric statistical methods.

The following statistical methods were used for the analysis of seizure parameters: comparison between the SE and PBN+SE groups was done using the non-parametric Mann-Whitney test. Comparison between PBN/PILO and PILO/PBN groups was done using the unpaired *t*-test. Multiple comparisons among all four groups were done using either the nonparametric Kruskal-Wallis test (H (number of groups) value and *p* value) followed by Dunn’s *post hoc* test or parametric one-way analysis of variance (ANOVA; F (DFn, DFd) value and *p* value) followed by Holm-Sidlak *post hoc* test. Comparison of the incidence of generalized tonic-clonic seizures during SE between the SE and PBN+SE groups was done using Fisher’s exact test. A *p* value of less than 0.05 was considered significant.

For analyzing the anxiety index, the resistance to capture, hippocampal volume and the number of DCX-ir cells, multiple comparisons among all four groups were done using the nonparametric Kruskal-Wallis test (H (number of groups) value and *p* value) followed by Dunn’s *post hoc* test.

To analyze hippocampal thickness, CA1 thickness, the number of hilar neurons, Prox1-ir cells and Prox1/NeuN-ir cells, multiple comparisons among all four groups were done with parametric one-way ANOVA (F (DFn, DFd) value and *p* value) followed by Holm-Sidlak *post hoc* test.

The data sets from the MWM test were analyzed by two-way repeated-measure ANOVA with one treatment factor (control, PBN, SE and SE+PBN) and session as a repeated-measure factor (five sessions). The *post hoc* Student-Newman-Keuls Method was used to analyze the latencies to reach the platform and the number of errors for each individual session. Data were analyzed using SigmaStat^®^ (SPSS Inc., Chicago, IL, USA) software. The level of significance was set at *p* < 0.05.

In the text, parametric data are presented as mean ± standard deviation (SD) and non-parametric data are presented as median with 25–75 percentile. In the graphs, parametric data are presented in bar graphs with error bars indicating SD. Nonparametric data are presented in box plots (median with 25–75 percentile) with whiskers indicating minimal and maximal values and symbols indicating values for each subject. In the figure legends, details are given concerning which tests were used and their results.

## Results

### PBN/PILO and PILO/PBN Groups

Statistical analysis revealed no difference between the PILO/PBN and PBN/PILO treatments in any measurement (Supplementary Table S1). Therefore, in the ensuing results, data from these two groups have been combined into a single group labeled PBN+SE.

In addition, for technical reasons, some animals were lacking data for one or more of the behavioral, electrophysiological or anatomical studies presented below. The absence of these data is reflected in the numbers of animals presented in the various sections of the results.

### Status Epilepticus

The parameters of SE were not significantly affected by PBN treatment. In the SE group, all 12 animals developed convulsive SE. Generalized tonic-clonic seizures occurred in four rats, all of which died. The eight surviving animals all exhibited a severity score of 3. The latency to SE onset was 1,185 ± 715 s. In the PBN+SE group, SE developed in 21 of the 23 animals and, like the untreated animals, they all had a severity score of 3. The latency to SE onset was 1,589 ± 856 s (Mann-Whitney, *U*_(183, 447)_ = 92; *p* = 0.085) compared to the SE group. In contrast to the SE group, none of PBN+SE animals died or exhibited generalized tonic-clonic seizures (Fisher’s test two-sided; *p* = 0.012).

### Long-Term Effects of PBN Treatment on Behavior

#### Elevated Plus Maze (Figure 2)

SE animals tended to be less anxious than animals experiencing no SE (SE vs. C or PBN in Figure 2B), spending significantly more time in the open arms. This result was reversed by PBN treatment (Kruskal-Wallis with Dunn’s *post hoc* test; *H*_(4)_ = 13.36; *p* = 0.0039). PBN+SE animals were significantly more anxious than the SE animals, spending more than twice as much time in the closed arms. Furthermore, PBN+SE animals tended to be more anxious than controls with 8 of the 19 PBN+SE animals never venturing into the open arms of the maze, whereas all animals in the other groups explored both the open and closed arms. Statistically, however, the anxiety index of the PBN+SE group did not differ from that of the control animals (C or PBN). This comparison, however, is complicated by the ceiling effect among the PBN+SE animals.

In the absence of SE, PBN treatment had no effect on the anxiety index (C vs. PBN in Figure 2B).

#### Resistance to Capture (Figure 3)

PBN treatment did not reverse the effects of SE on aggressiveness. In fact, PBN+SE animals were significantly more aggressive than the SE animals (Kruskal-Wallis with Dunn’s multiple comparison; *H*_(4)_ = 39.22; *p* < 0.0001). SE animals resisted capture more vigorously than controls, but increase of handling score was not significant. The PBN+SE group surpassed this level significantly (C – 1 (1, 1.25); PBN – 1 (1, 1); SE – 2 (2, 2.75); PBN+SE 4 (3, 4); Kruskal-Wallis with Dunn’s *post hoc* test; *H*_(4)_ = 39.22; *p* < 0.0001). What is even more striking is that 10 of the 21 PBN+SE rats had a maximum handling score of 4, while none of the animals in any of the other groups did.

#### Morris Water Maze Test (Figure 4)

The most notable result of the MWM data was the inability of the SE animals to learn the task. Their latencies and their number of failures were essentially unchanged across the five sessions. By contrast, both control groups readily learned the task with improved latencies and decreased failures across the sessions. Interestingly, animals treated with PBN alone actually outperformed the C animals during the first two sessions.

PBN treatment partially reversed the effects of SE. The PBN+SE animals performed substantially better the SE animals. From session 3 onward, they had shorter latencies (two way repeated measure ANOVA with *post hoc* Kruskal-Wallis revealed treatment (two-way ANOVA; *F*_(3,168)_ = 10.68, *p* < 0.001), session (*F*_(4,168)_ = 70.84, *p* < 0.001) and treatment × session interaction (*F*_(12,168)_ = 5.26, *p* < 0.001) effects) and fewer failures (two way repeated measure ANOVA with* post hoc* Kruskal-Wallis revealed treatment (*F*_(3,168)_ = 14.75; *p* < 0.001), session (*F*_(4,168)_ = 63.72; *p* < 0.001) and treatment × session interaction (*F*_(12, 168)_ = 6.22; *p* < 0.001) effects. On the other hand, the PBN+SE animals did not perform as well as the PBN controls during any session with consistently longer latencies and more errors. In addition, the cumulative latency for the PBN+SE group was significantly longer than that for the PBN group (Kruskal-Wallis with Dunn’s multiple comparison; *H*_(4)_ = 20.47; *p* < 0.0001).

### Long-Term Effects of PBN Treatment on Seizure Parameters (Figure 5)

The results of PBN treatment on the development of chronic epilepsy was the most surprising outcome of this study. Rather than ameliorate the long term effects of SE, PBN treatment actually exacerbated seizures 4–5 months after SE.

In the PBN+SE group, seizure frequency (mean number of seizures per 24 h) was 6.1 times higher than in the SE group (Mann-Whitney RST; *U*_(44,127)_ = 16; *p* = 0.0441; Figure 5A). In addition, the mean seizure duration per 24 h was significantly longer in the PBN+SE group (Mann-Whitney RST; *U*_(39,132)_ = 11; *p* = 0.0114; Figure 5B). These animals also had increased seizure activity, spending significantly more total time seizing during a 24 h period than the SE group (Mann-Whitney RST; *U*_(43,128)_ = 15; *p* = 0.0346; Figure 5C). In both the PBN+SE and the SE groups more than 80% of the seizures (90.8% and 83.1%, respectively) had electrical seizure activity spread beyond the hippocampus to the cerebral cortex. All of these seizures were associated with behavioral correlates (stages 3–5 according to Racine’s scale; Racine, 1972).

### Long-Term Effects of PBN Treatment on Morphology of the Hippocampus (Figures 6, 7)

Administration of PBN during SE resulted in partial protection against structural damage in the hippocampus (Figure 6B). Untreated SE resulted in significant damage to the hippocampus: the thickness of the septal hippocampus was lower by 17.2% (one-way ANOVA following Holm-Sidak’s *post hoc* test; *F*_(3,37)_ = 7.677; *p* = 0.0004) and the thickness of the CA1 was lower by 35.8%. In the PBN+SE group, hippocampal thickness was significantly increased in comparison to the SE group (one-way ANOVA with *post hoc* Holm-Sidak’s test; *F*_(3,37)_ = 16.8; *p* < 0.0001) and the results in the PBN+SE animals did not differ statistically from the PBN controls. In CA1, PBN treatment increased hippocampal thickness in comparison to SE animals, but the CA1 subregion in the PBN+SE animals was still significantly thinner than in the PBN controls. Administration of PBN alone did not affect any measured parameter of hippocampal morphology.

PBN treatment significantly protected the hippocampus from overall volume loss caused by SE. SE significantly reduced the mean volume of the hippocampus by 28.7% in comparison to the C group. The mean hippocampal volume of the PBN+SE group was no different from that of either control group, although the volumes varied considerably among the PBN+SE animals (Kruskal-Wallis with *post hoc* Dunn’s test; H_(4)_ 11.41; *p* = 0.0097; Figure 6C).

Finally, PBN treatment partially protected against SE-induced loss of hilar neurons (Figure 6D). The PBN+SE animals had significantly more hilar neurons than the SE animals, but the number of neurons in the PBN+SE group was still lower than that of the control groups (one-way ANOVA with *post hoc* Holm-Sidak’s test; *F*_(3,37)_ = 14.57; *p* < 0.0001).

### Long-Term Effects of PBN Treatment on Hippocampal Neurogenesis

The effect of PBN treatment on aberrant neurogenesis in the DH was another surprising outcome of the present study. Strikingly, PBN+SE animals had significantly more cells expressing both Prox1 and NeuN in comparison to SE animals (Figure 8B). In both control groups, only sparse cells co-expressing Prox1 and NeuN were observed in each evaluated section (215 ± 31.5 in the C group and 273 ± 78.5 in the PBN group; data expressed as mean ± SD). Following SE alone, these numbers were significantly increased by more than 10 times. PBN treatment at the time of SE further increased the number of Prox1/NeuN-ir cells by 51.1% (one-way ANOVA with *post hoc* Holm-Sidak’s test; *F*_(3,37)_ = 27.34; *p* < 0.0001).

By contrast, the number of cells expressing Prox1 alone was not affected by PBN treatment. SE markedly increased the number of these aberrant cells in the hilus, but the addition of PBN did not significantly change the count. In both control groups, only a few cells that met the criteria for Prox1-ir cells were present in each evaluated section (500 ± 59 in the C group and 541 ± 183 in PBN controls; data expressed as mean ± SD). SE increased the number of these cells 25 times in the SE group and 24 times in the PBN+SE group (one-way ANOVA with *post hoc* Holm-Sidak’s test; *F*_(3,37)_ = 8.208; *p* = 0.0003; Figure 8A).

**Figure 9:**
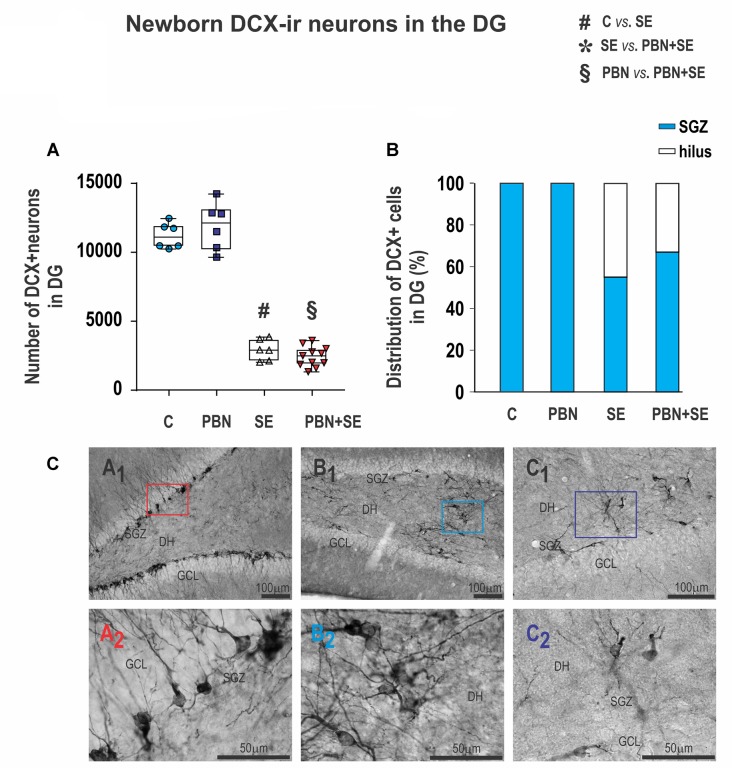
Long term effect of PBN treatment on newborn DXC-ir cells in the DG. **(A)** Numbers of DXC-ir cells observed in the DG of individual animals in each group. SE, with or without PBN treatment, significantly suppressed neurogenesis. Data presented as box plots (median with 25%–75%) with whiskers indicating minimal and maximal values. **(B)** Distribution of DXC-ir cells between the subgranular zone (SGZ) and the hilus of the DG. In control animals, all the cells are located in the SGZ, while in animals receiving SE, substantial numbers of the cells are ectopically located in the hilus. **(C)** Representative photomicrographs of DXC-ir cells in the DG. **(A1)** Control animal showing large numbers of DXC-ir cells exclusively located in the SGZ. **(A2)** Higher magnification of box in **(A1)**. **(B1,C1)** SE animals showing reduced numbers of DXC-ir cells that are distributed both in the SGZ and in the hilus. **(B2,C2)** Higher magnifications of boxes in B1 and C1, respectively, showing that the aberrant cells in the hilus have abnormal bipolar morphology and dense basal dendrites (Abbreviations same as in Figure 2).

PBN treatment also did not affect the number of newborn cells in the hippocampus. The results were essentially the same in SE and PBN+SE animals. In both groups, SE dramatically reduced the number of DCX-ir cells in comparison to controls (Kruskal-Wallis with *post hoc* Dunn’s test; H_(4)_ 21.27; *p* < 0.0001; Figure 9A).

Furthermore, the location of DCX-ir cells was significantly affected by SE. In both control groups, DCX-ir cells were located exclusively in the dentate GCL and the dentate SGZ (Figures 9CA1,A2). By contrast, significant numbers of newborn neurons were located ectopically in the hilus in both the SE (45%) and PBN+SE animals (33%; Figure 9B). The remaining DCX-ir cells were located in the SGZ. Each evaluated section usually contained only a few DCX-ir cells in the SGZ, either isolated or in small clusters separated by large gaps. A substantial number of newborn neurons, particularly those located in the DH, were bipolar or displayed dense basal dendrites (Figures 9CB1,B2,C1,C2).

## Discussion

The present study addressed the question: does free radical scavenging protect against the harmful long-term consequences of SE in immature animals? Surprisingly, we found that the answer was complex.

We used PBN as a model free radical scavenger. PBN was administered to P25 rats at the time of SE induced by LiCl/PILO. Later in life these animals exhibited variable outcomes from this treatment. Some were beneficial and some actually made the effects of SE worse.

On the beneficial side, PBN treatment protected against the structural damage in the hippocampus known to occur as a consequence of SE (Nitecka et al., 1984; Lothman and Bertram, 1993; Sankar et al., 1998). We have shown previously that PBN has a significant neuroprotective effect acutely (Rejchrtová et al., 2005) and the present results demonstrated long-term protection, although not complete. Hippocampal thickness, subfield CA1 thickness and hippocampal volume were all statistically greater in PBN treated animals than in animals subjected to SE alone. Nevertheless, the PBN+SE animals still had a statistically thinner CA1 subsection than control animals. Furthermore, the degree of preservation of hippocampal structure varied widely among the treated animals.

PBN treatment also had a beneficial effect on performance in the MWM, but again, the preservation was only partial. PBN+SE animals performed significantly better than animals subjected to SE without treatment. In fact, the SE animals were completely unable to learn the task, whereas the PBN treated animals steadily improved their performance in the maze with each session. Nevertheless, the PBN+SE animals were significantly slower in learning the task than the control groups and continued to make errors even in the final fifth session, while the controls were error-free after the fourth session.

Our results support previous studies that demonstrated that the extent of hippocampal damage correlates with the severity of spatial memory deficits (Broadbent et al., 2004; Kubová and Mareš, 2013; Maia et al., 2014). In addition, it has been shown that other neuroprotective therapies that reduce hippocampal neuronal loss also improve behavior in the MWM (Block and Schwarz, 1997).

Our results do not support previous findings suggesting a negative correlation between performance in the MWM and seizure frequency (Nissinen et al., 2000; Majak and Pitkänen, 2004). The PBN treated animals in our study exhibited about six times more seizures than the non-treated animals, but performed significantly better at the task. It must be pointed out, however, that seizure frequency in our study was formally assessed nearly 2 weeks after testing in the MWM. Therefore it is possible that the occurrence of seizures was different at the time of behavioral assessment. Nevertheless, informal observations of the animals during behavioral testing did not give any indication of such a discrepancy.

On the negative side, PBN treatment worsened the long term outcome for other behavioral tests. In accord with a previous report by Detour et al. (2005), animals undergoing SE were less anxious than controls in the EPM. This effect was reversed by PBN, but the treated animals were not normalized. Rather, the behavior of PBN+SE animals was markedly different from that of controls. The treated animals appeared to be more anxious, with 8 of the 19 rats spending the entire test in an enclosed arm (minimal anxiety index score of 0). This result sharply contrasted with all the other groups where all the animals spent time in both arms. Statistically, the PBN+SE group did not differ from either of the control groups, but the marked ceiling effect observed in the PBN+SE animals complicates a statistical comparison.

The effect of PBN on aggressiveness also was negative. In accord with previous studies (Huang et al., 2012), SE significantly increased aggressive behaviors in the capture test. Again, however, rather than normalizing this behavior, PBN treatment actually exacerbated aggressive behaviors. Ten of the 21 PBN+SE animals received the maximum score of 4 in the capture test and the remaining 11 animals scored a 3. None of the SE animals scored a 4 and only two of eight scored a 3 with the rest scoring a 2.

Thus, far from protecting against the long-term effects of SE on anxiety and aggressiveness, free radical scavenging with PBN actually produced more detrimental long-term outcomes.

Also contrary to the expectation of neuroprotection, PBN treatment during SE had a detrimental long-term effect on the development of chronic epilepsy. The treated animals developed more severe epilepsy by all measures. The PBN+SE animals had significantly increased seizure frequency, seizure duration and seizure activity in comparison to SE animals. These results are surprising given that previous studies targeting the removal of reactive oxygen species (ROS) at the time of SE have not shown such effects.

Antioxidant administration at the time of SE has demonstrated either no effect on long-term seizure activity (Pearson et al., 2015) or a reduction in the frequency of spontaneous seizures at 5 months (Pauletti et al., 2017). These studies employed different antioxidant agents as well as different protocols for their administration in relationship to SE, which probably accounts for the different results.

Indeed, recent studies have pointed out that ROS have a complex relationship to epileptogenesis, perhaps involving neuroinflammation (see McElroy et al., 2017). How and when these processes act to influence seizure susceptibility following SE are unknown, but they probably extend beyond the acute phase of SE. Therefore, it is not surprising that different protocols to attack ROS and oxidative stress can have different outcomes. An optimal protective methodology has yet to be developed.

Regardless, to our knowledge, no study attempting to protect against the development of epilepsy following SE by free radical scavenging has shown long-term exacerbation of seizure activity as we have demonstrated in the present article. The question then arises: what mechanism might account for our present results?

Older literature explored the role of hippocampal neuronal loss in the development of chronic epilepsy. This line of inquiry, however, has not proven to be particularly fruitful with controversial results. Some studies have suggested a correlation between neuronal loss and seizure activity (for review see Lothman and Bertram, 1993; Wasterlain et al., 1993, 1996; Lowenstein, 1996), while other studies have failed to find any such correlation (Sloviter, 1992; Buckmaster and Dudek, 1997; Pitkänen et al., 2002; Zhang et al., 2002).

More recently, new lines of inquiry have suggested that the development of chronic epilepsy following SE is related to rewiring of hippocampal circuitry due to aberrant neurogenesis. In particular, it has been demonstrated that following an epileptogenic insult, such as SE, newborn cells destined to become GC neurons do not always migrate correctly to the GCL of the dentate gyrus (DG). Rather, some cells migrate aberrantly into the DH (Parent et al., 2006; Scharfman et al., 2007), where they can persist (Jessberger et al., 2007).

Furthermore, electrophysiological studies in hippocampal slices from rats with chronic epilepsy suggest that these ectopic cells integrate into dentate circuitry and have a major impact on the excitability of the hippocampal network (Scharfman et al., 2003). They receive more excitatory inputs and promote increased hippocampal excitability (Myers et al., 2013). On the other hand, adult born GCs that correctly migrate to the GCL show decreased excitability (Jakubs et al., 2006) or a maintenance of excitability (Wood et al., 2011). Thus, the data suggest that there are two populations of adult born GCs following SE: an aberrant population in the hilus that induces hyperexcitability and a normal population in the GCL that tends to restore inhibition. The balance between these two populations may be a major factor in determining the development of recurrent seizures following SE.

The balance between the excitatory and inhibitory effects induced by adult neurogenesis is likely to be very complex, however. For example, Jung et al. (2004, 2006) showed that treatment of animals with a cell proliferation inhibitor at the time of an epileptogenic brain injury reduced the frequency of spontaneous seizures later in life. On the other hand, Hosford et al. (2016) found that GC ablation after SE reduced the frequency of spontaneous seizures while paradoxically increasing seizure duration. Iyengar et al. (2015) suppressed neurogenesis before SE and showed that this treatment actually enhanced the sensitivity to chemoconvulsant agents. Finally, Cho et al. (2015) demonstrated that the severity of chronic epilepsy was reduced if neurogenesis was ablated 4 weeks prior to SE, but the same procedure done immediately before or after SE was ineffective. These findings suggest that the degree of development of newborn cells may be important in the relationship between adult neurogenesis and epilepsy.

Our present findings tend to support the hypothesis that the aberrant population of neurons occurring in the hilus following SE may contribute significantly to the development of chronic epilepsy. We confirmed that SE in juvenile animals leads to the appearance of ectopic Prox1-ir GCs in the DH, and that the number of these Prox1-ir cells correlates with the severity of seizure activity. McCloskey et al. (2006) demonstrated a similar positive association between the number of Prox1-ir cells in the DH and seizure frequency in rats experiencing SE as adults.

On the other hand, the number of aberrant hilar Prox1-ir cells was essentially the same in PBN+SE animals and SE animals, not reflecting the increased seizure severity in the PBN+SE group. This discrepancy means that some other factor must be contributing to the increased seizure severity following PBN treatment. Our data suggest that this added factor is the state of maturity of the aberrant hilar GCs.

Prox1 is a specific marker for cells destined to become GCs but it is expressed not only in mature neurons but also in immature cells that are still differentiating (Torii et al., 1999). Thus, Prox-1 immunoreactivity labels a heterogeneous mixture of cells. All of them are in the GC line but they are at different stages of development. To examine the state of maturity of aberrant hilar GCs, we labeled cells with a second antibody that reacts with NeuN, a nuclear protein that is specific for mature neurons. Thus, we defined a subset of double-labeled mature GC neurons aberrantly located in the hilus.

We found significantly more double-labeled neurons in animals treated with PBN than in untreated SE animals. Thus, PBN treatment caused a greater number of mature GC neurons to become ectopically located in the hilus. If one postulates that only these mature ectopic neurons are capable of functionally integrating into hippocampal circuitry, then the greater seizure severity of PBN+SE animals is potentially explained. The presence of greater numbers of mature GC neurons in the DH of PBN-treated rats would produce a greater degree of aberrant excitatory circuitry in their hippocampi. This aberrant circuitry, in turn, would render these animals more epileptic.

Our study also confirms the finding of Hattiangady et al. (2004) that in the chronic epileptic state there is markedly diminished neurogenesis in the hippocampus. We found a dramatic decline in the production of cells expressing DCX, a marker of neuronal progenitor cells, in SE and PBN+SE animals. It has been estimated that DCX is expressed up to a maximum of 12 days after the birth of newborn neurons (Rao and Shetty, 2004). Thus, the majority of the DCX-ir cells identified in our study were born during the time of video/EEG monitoring when it was demonstrated that the animals were having recurrent spontaneous seizures.

It has also been suggested that this suppression of neurogenesis plays a significant role in the maintenance of chronic epilepsy (Hattiangady and Shetty, 2008). While these authors acknowledge that there is no direct evidence to support this suggestion, there are significant hints. For example, Liu et al. (2003) have shown that ~14% of newborn neurons in the DG of young rats differentiate into inhibitory GABAergic cells. Thus, the lack of neurogenesis in chronic epilepsy could result in reduced inhibitory circuitry in the DG, removing a potential counterbalance to the hyperexcitability produced by aberrant circuitry in the hilus.

Furthermore, our data show a negative correlation between the number of DCX-ir cells and seizure severity. In particular, there were 17% fewer DCX-ir in PBN+SE animals. This finding raises the possibility that the greater degree of seizure activity is due not only to hyperexcitable aberrant circuitry in the hilus, but also to a lack of counterbalancing inhibition in the DG.

The role of aberrant neurogenesis in the behavioral and cognitive comorbidities of chronic epilepsy is more difficult to determine. To our knowledge, there are no direct data suggesting an association between emotional impairment and the type of aberrant neurogenesis we have demonstrated in the present study. Nevertheless, there are some indirect data bearing on this association.

Hester and Danzer (2014) reported that animals with genetically disrupted adult neurogenesis, leading to abnormal GCs, exhibit a variety of behavioral abnormalities, including cognitive deficits, increased anxiety and depressive and schizophrenic-like behavior. Other studies have demonstrated that suppression of adult neurogenesis improves epilepsy-related cognitive decline (Jessberger et al., 2007; Pekcec et al., 2008; Cho et al., 2015). Furthermore, Hattiangady and Shetty (2008), whom we cited earlier for their suggestion that suppression of neurogenesis might play a significant role in the maintenance of chronic epilepsy, have also suggested that a reduction of normal neurogenesis may be important in epilepsy comorbidities.

Altered neurogenesis has also been observed in animal models of behavioral pathology. Malberg and Duman (2003) reported decreased neurogenesis in a model of depression. Moreover, treatment with antidepressant medication increased neurogenesis (Malberg et al., 2000) and suppression of neurogenesis by stress was found to block the effects of antidepressant therapy (Malberg and Duman, 2003).

Our own data in the present study show a strong negative correlation between the number of Prox1/NeuN-ir cells and the anxiety index. This finding, however, must be interpreted with caution for two reasons. First, mathematical correlation does not prove causality. Second, the neuronal network underlying emotionality is very complex and involves many other structures besides the hippocampus known to be damaged after SE (e.g., amygdala and thalamic nuclei).

In conclusion, the present study has shown that the administration of the free radical scavenger, PBN, during SE in juvenile rats modifies the long-term consequences of this insult in varying ways, both beneficial and detrimental. PBN beneficially protected against the severe anatomical damage in the hippocampus and associated spatial memory impairment. On the other hand, animals treated with PBN developed more severe epilepsy and emotional deficits later in life than did animals that experienced SE alone. The mechanisms underlying these effects of PBN are most likely complex, but our data suggest the working hypothesis that a significant aspect of PBN’s action is to modify postnatal neurogenesis in the hippocampus. SE acutely increases neurogenesis and aberrant migration of GCs to the DH. PBN appears to exacerbate this process. In particular PBN results in increased numbers of mature hilar GCs that may abnormally integrate into hippocampal circuitry and produce both excessive excitability and behavioral comorbidities. Concurrently, PBN also appears to exacerbate the suppression of normal neurogenesis in the DG caused by chronic epilepsy. This action potentially eliminates a counterbalancing inhibitory force in the hippocampus.

Aberrant neurogenesis is a most intriguing explanation for our findings, but it must be acknowledged that this not the only possible mechanism. Other explanations include specific areas of neuronal loss or damage induced by SE accompanied by specific cell populations protected by the action of PBN as described in some conventional antiepileptic drugs before (for review see Pitkänen and Kubová, 2004). For example, Peterson et al. (2005) demonstrated that PBN provided acute protection (24 h after Li/PILO-induced SE) in parietal, occipital, perirhinal and piriform cortices as well as lateral amygdala. By contrast, PBN exacerbated neuropathology in CA3 and thalamus.

These widespread changes could lead to synaptic reorganization and rewiring of the brain. Such actions may be especially important in juvenile animals that are in the process of developing the balance between excitation and inhibition and cognitive and developing cognitive and emotional functions.

## Author Contributions

All authors have made substantial, intellectual and direct contribution to the work, and approved it for publication. HK, JF, JB and PM conceived and designed the experiments, JR, MP, AM and GT performed the experiments and analyzed the data. HK and JB wrote the manuscript.

## Conflict of Interest Statement

The authors declare that the research was conducted in the absence of any commercial or financial relationships that could be construed as a potential conflict of interest.

## Footnotes

^1^https://www.nc3rs.org.uk/arrive-guidelines

^2^https://www.nc3rs.org.uk/the-3rs

## Abbreviations

DCX: doublecortin
EPM: elevated plus maze
MWM: Morris water maze
NeuN: neuronal specific nuclear protein
PBN: N-tert-butyl-α-phenylnitrone
PILO: pilocarpine
Prox-1: prospero-like homeobox protein 1
TLE: temporal lobe epilepsy.
