# Supplementary material for: The Free Radical Scavenger N-Tert-Butyl-α-Phenylnitrone (PBN) Administered to Immature Rats During Status Epilepticus Alters Neurogenesis and Has Variable Effects, Both Beneficial and Detrimental, on Long-Term Outcomes
Source: Front Cell Neurosci. 2018 Aug 28;12:266. doi: 10.3389/fncel.2018.00266 (PMC6121067; doi:10.3389/fncel.2018.00266)
Supplement: Supplementary file 1 [file Table_1.DOCX]

**Supplementary Table S1**: Comparison between two treatment groups (PILO/PBN and PBN/PILO) was done using unpaired two-tailed t-test. Data presented as mean±SD

| ***Parameter*** | **PBN/pilo** | **Pilo/PBN** | **Unpaired two-tailed t-test** |
| --- | --- | --- | --- |
| Hippocampal thickness | 1.654±0.111 mm | 1.692±0.198 mm | t=0.53, df=18, *p=0.604* |
| CA1 thickness | 0.631±0.154 mm | 0.576±0.082 mm | t=1.011  df=18,  *p=0.325* |
| Anxiety index | 26.3±26.1 % | 16.5±27.9 % | t=0.794  df=17,  *p=0.438* |
| Resistance to capture (score) | 3.4±0.7 | 3.3±0.8 | t=0.39  df=19,  *p=0.700* |
| Cumulative latency (MWM) | 755±303 s | 649±281 s | t=0.7428  df=15,  *p=0.469* |
| Seizure frequency (seizures/24h) | 30.6±40.2 | 27.5±30.1 | t=0.1456  df=9,  *p=0.887* |
| Seizure activity (total seizure duration per 24 h) | 918.9±1258 s | 1210.6±1385 s | t=0.3621  df=9,  *p=0.726* |
| Hippocampal volume (mm3) | 50.0±8.3 mm^3^ | 54.1±8.1 mm^3^ | t=1.011  df=18,  *p=0.268* |
| Hilar neurons | 13706±4392 | 15395±3155 | t=1.001  df=18,  *p=0.330* |
| Prox1-ir | 9609±3784 | 14399±7088 | t=1.623  df=15,  *p=0.125* |
| Prox1/NeuN-ir | 3335±973 | 3865±1808 | t=0.6524  df=13,  *p=0.525* |
| Doublecortin-ir (total number) | 2429±966 | 2434±440 | t=0.009  df=9,  *p=0.992* |
